# Supplementary material for: Dynamic transcriptomic profiles of zebrafish gills in response to zinc supplementation
Source: BMC Genomics. 2010 Oct 11;11:553. doi: 10.1186/1471-2164-11-553 (PMC3091702; doi:10.1186/1471-2164-11-553)
Supplement: Additional file 2 — Interactive Direct Interaction Network representing the molecular interactions between zinc, copper, iron, calcium and proteins encoded by transcripts changed by zinc supplementation. Mini web-site containing index.html and hyperlinked pages in subdirectory describing a Direct Interaction Network automatically generated based on curated interactions contained within the proprietary PathwayArchitect database. Ovals represent proteins and the circles symbolize metal ions. Objects are coloured by their abundance in zebrafish at the time-point they were significantly different from the control is a scale from -4 fold (dark green) to +4 fold (dark red). Where significant differences were found at more than one time-point, the colour overlay shows expression at the first instance. Dark blue squares denote 'binding', and light blue squares 'expression'; green squares stand for 'regulation', green diamonds for 'metabolism', and green circles for 'promoter binding'. Arrow heads indicate directionality of the interaction where annotated. All nodes and edges can be further interrogated by selecting the relative area of the image. [file 1471-2164-11-553-S2.zip › PathwayArchitect Zn xs DIN/151772.html]

# PROTEIN: COL1A1

|  |  |
| --- | --- |
| Name | COL1A1 |
| Type | PROTEIN |
| Description | collagen, type I, alpha 1 |
| Note | This gene encodes the major component of type I collagen, the fibrillar collagen found in most connective tissues, and the only component of the collagen found in cartilage. Mutations in this gene are associated with osteogenesis imperfecta, Ehlers-Danlos syndrome, and idiopathic osteoporosis. Reciprocal translocations between chromosomes 17 and 22, where this gene and the gene for platelet-derived growth factor beta are located, are associated with a particular type of skin tumor called dermatofibrosarcoma protuberans, resulting from unregulated expression of the growth factor. Two transcripts, resulting from the use of alternate polyadenylation signals, have been identified for this gene. |
| Alias | Collagen alpha 1(I) chain precursor |
|  | COLIA1 |
|  | Collagen alpha 1 chain |
|  | procollagen type I alpha 1 |
|  | alpha 1 type I collagen |
|  | alpha-1 type I collagen |
|  | aa 694-711 |
|  | Mov13 |
|  | osteogenesis imperfecta type IV |
|  | collagen of skin, tendon and bone, alpha-1 chain |
|  | collagen alpha 1 chain type I |
|  | Col1a-1 |
|  | COL1A1 |
|  | Fragments |
|  | Col1a1 |
|  | alpha-1 type 1 collagen |
|  | type I collagen alpha 1 chain |
|  | pro-alpha-1 collagen type 1 |
|  | type II procollagen gene fragment |
|  | Cola-1 |
|  | Collagen I, alpha-1 polypeptide |
|  | Cola1 |
|  | type I collagen pro alpha 1(I) chain propeptide |
|  | procollagen type I, alpha 1 |
|  | OI4 |
|  | Mov-13 |


---

|  |  |
| --- | --- |
| GO Component | collagen |
|  | extracellular space |
|  | extracellular matrix (sensu Metazoa) |
|  | collagen type I |
|  | cytoplasm |


---

|  |  |
| --- | --- |
| GO ID | GO:0005584 |
|  | GO:0005615 |
|  | GO:0005201 |
|  | GO:0005737 |
|  | GO:0005578 |
|  | GO:0006817 |
|  | GO:0007155 |
|  | GO:0030020 |
|  | GO:0008544 |
|  | GO:0007605 |
|  | GO:0005198 |
|  | GO:0005581 |
|  | GO:0001501 |
|  | GO:0008147 |


---

|  |  |
| --- | --- |
| MIM | MIM:114000 |
|  | MIM:130000 |
|  | MIM:259420 |
|  | MIM:166210 |
|  | MIM:166220 |
|  | MIM:130060 |
|  | MIM:166200 |
|  | MIM:166710 |
|  | MIM:120150 |


---

|  |  |
| --- | --- |
| Connectivity | 523 |


---

|  |  |
| --- | --- |
| Entrez ID | 29393 |
|  | 1277 |
|  | 12842 |


---

|  |  |
| --- | --- |
| Agilent ID | A\_14\_P115885 |
|  | A\_51\_P377094 |
|  | A\_53\_P107733 |
|  | A\_44\_P419137 |
|  | A\_23\_P207521 |
|  | A\_53\_P105527 |
|  | A\_53\_P132500 |
|  | A\_52\_P658611 |
|  | A\_51\_P377095 |
|  | A\_52\_P525107 |
|  | A\_23\_P207520 |


---

|  |  |
| --- | --- |
| Cellular Localization | Cytoplasm |
|  | Extracellular matrix |
|  | Extracellular region |
|  | Cell |


---

|  |  |
| --- | --- |
| DbXref | KEGG pathway##04512##ECM-receptor interaction##http://www.genome.jp/dbget-bin/show\_pathway?hsa04512+1277 |
|  | KEGG pathway##04510##Focal adhesion##http://www.genome.jp/dbget-bin/show\_pathway?hsa04510+1277 |
|  | KEGG pathway##04512##ECM-receptor interaction##http://www.genome.jp/dbget-bin/show\_pathway?mmu04512+12842 |
|  | Reactome##109582##Hemostasis##http://www.reactome.org/cgi-bin/eventbrowser?DB=gk\_current&ID=109582 |
|  | KEGG pathway##04510##Focal adhesion##http://www.genome.jp/dbget-bin/show\_pathway?mmu04510+12842 |


---

|  |  |
| --- | --- |
| Pathway | Zn xs inventory |
|  | Zn xs DIN |


---

|  |  |
| --- | --- |
| GO Process | cell adhesion |
|  | phosphate transport |
|  | skeletal development |
|  | epidermis development |
|  | perception of sound |


---

|  |  |
| --- | --- |
| UniGene | Rn.2953 |
|  | Mm.277735 |
|  | Hs.172928 |


---

|  |  |
| --- | --- |
| Affymetrix Probeset ID | 1370864\_at |
|  | 1388116\_at |
|  | 1423669\_at |
|  | 1556499\_s\_at |
|  | 202310\_s\_at |
|  | 202311\_s\_at |
|  | 202312\_s\_at |
|  | 35473\_at |
|  | 35474\_s\_at |
|  | 94305\_at |
|  | g4502944\_3p\_a\_at |
|  | Hs.172928.0.A1\_3p\_a\_at |
|  | Hs.172928.0.A2\_3p\_a\_at |
|  | Hs.172928.1.A1\_3p\_a\_at |
|  | Hs2.433191.1.A1\_3p\_s\_at |
|  | M27207mRNA\_s\_at |
|  | M55998\_s\_at |
|  | Msa.117.0\_f\_at |
|  | rc\_AI231472\_s\_at |
|  | u08020\_f\_at |
|  | U75405UTR#1\_f\_at |
|  | Z74615\_at |
|  | Z78279\_at |
|  | Z78279\_g\_at |
|  | 103709\_at |
|  | 1455494\_at |
|  | 217430\_3p\_x\_at |
|  | 217430\_x\_at |
|  | aa562685\_f\_at |
|  | TC16669\_s\_at |
|  | TC19462\_s\_at |
|  | TC22866\_f\_at |
|  | TC33957\_i\_at |
|  | TC33957\_s\_at |
|  | TC39339\_f\_at |
|  | TC40853\_f\_at |
|  | TC40854\_at |


---

|  |  |
| --- | --- |
| GO Function | extracellular matrix structural constituent |
|  | structural molecule activity |
|  | extracellular matrix structural constituent conferring tensile strength |
|  | structural constituent of bone |


---

|  |  |
| --- | --- |
| Nucleotide | M24372 |
|  | M12199 |
|  | X00299 |
|  | AK159285 |
|  | XM\_213440 |
|  | AK158011 |
|  | AL662790 |
|  | M36546 |
|  | U38544 |
|  | K03032 |
|  | AK159283 |
|  | X54876 |
|  | AK159300 |
|  | M60424 |
|  | J00112 |
|  | X06753 |
|  | L47667 |
|  | M32798 |
|  | K03029 |
|  | S64596 |
|  | BC036531 |
|  | S67530 |
|  | NM\_007742 |
|  | M23213 |
|  | U08020 |
|  | K03033 |
|  | S58915 |
|  | M20789 |
|  | K01228 |
|  | M55998 |
|  | K03036 |
|  | M14423 |
|  | M10627 |
|  | X06269 |
|  | AB209597 |
|  | Z74615 |
|  | AF017178 |
|  | AK178289 |
|  | K03030 |
|  | AL606480 |
|  | J03559 |
|  | J00111 |
|  | BC003198 |
|  | M11162 |
|  | J02829 |
|  | X07884 |
|  | BC059281 |
|  | X57981 |
|  | Z78279 |
|  | X15896 |
|  | BC050014 |
|  | U03419 |
|  | M17491 |
|  | X00820 |
|  | K03035 |
|  | J00113 |
|  | K01688 |
|  | NM\_000088 |
|  | AK132180 |
|  | S67482 |
|  | J00110 |
|  | K03034 |
|  | M27208 |
|  | K03031 |
|  | K03179 |


---

|  |  |
| --- | --- |
| Protein | P02454 |
|  | AAH36531 |
|  | AAA37330 |
|  | AAA75386 |
|  | P11087 |
|  | AAA52052 |
|  | AAA80676 |
|  | AAB59363 |
|  | NP\_000079 |
|  | AAA52036 |
|  | AAA52049 |
|  | AAA60150 |
|  | NP\_031768 |
|  | AAA51992 |
|  | AAB59373 |
|  | BAD92834 |
|  | AAH03198 |
|  | AAA51847 |
|  | AAA88912 |
|  | AAN86574 |
|  | AAA51993 |
|  | AAB94054 |
|  | AAA64617 |
|  | CAB01633 |
|  | AAA37333 |
|  | CAA25394 |
|  | AAA37332 |
|  | AAB59576 |
|  | AAA37130 |
|  | CAA30731 |
|  | CAI23970 |
|  | AAH50014 |
|  | AAA52291 |
|  | CAA29927 |
|  | CAA38657 |
|  | AAA37334 |
|  | AAA03475 |
|  | XP\_213440 |
|  | AAA40834 |
|  | AAA37129 |
|  | AAH59281 |
|  | AAA51995 |
|  | CAA33904 |
|  | CAA29605 |
|  | AAA52289 |
|  | AAB29424 |
|  | AAB26324 |
|  | CAA41046 |
|  | AAA52290 |
|  | CAA98968 |
|  | AAB27856 |
|  | CAI25880 |
|  | P02452 |
|  | CAA25082 |


---

|  |  |
| --- | --- |
| Organism | Mammal |


---

|  |  |
| --- | --- |
| Location | chromosome 11, 11 56.0 cM, 11 D (Mus musculus) |
|  | chromosome 17, 17q21.3-q22.1 (Homo sapiens) |
|  | 11 56.0 cM (Mus musculus) |
|  | chromosome 10, 10q31 (Rattus norvegicus) |


---

|  |  |
| --- | --- |
